# Editorial: Emerging Ototoxic Medications and Their Role in Cochlear and Vestibular Disorders

**DOI:** 10.3389/fneur.2021.773714

**Published:** 2021-10-22

**Authors:** Agnieszka J. Szczepek, Kostantina M. Stankovic

**Affiliations:** ^1^Department of Otorhinolaryngology, Head and Neck Surgery, Charité-Universitätsmedizin Berlin, Corporate Member of Freie Universität Berlin and Humboldt-Universität zu Berlin, Berlin, Germany; ^2^Faculty of Medicine and Health Sciences, University of Zielona Gora, Zielona Gora, Poland; ^3^Department of Otolaryngology-Head and Neck Surgery, Stanford University School of Medicine, Stanford, CA, United States

**Keywords:** ototoxicity, cochlea, vestibular system, medications, COVID-19, tuberculosis, MDR–multi drug resistant

Ototoxicity is an adverse effect of medication that negatively affects the sense of hearing, balance, or both. Unfortunately, the negative effects of ototoxic drugs are often irreversible, permanently decreasing the quality of life of the affected individuals. Aspirin is one of the oldest medications, causing reversible ototoxicity that has been researched but is still not well-understood. As early as 1877, a case report described a patient with tinnitus resulting from treatment with up to 15 g a day of sodium salicylate (1). Also, acute intoxication with salicylate remains an issue today (2).

Generations of students have graduated from medical schools equipped with knowledge regarding the ototoxic properties of aspirin and three other classes of medications: aminoglycosides, platinum-containing cytostatics, and loop diuretics. Nevertheless, new drugs appear annually on the pharmacy shelves, and old and forgotten drugs are repurposed. In addition, ototoxicity caused by some medications develops slowly over many years. “*Emerging Ototoxic Medications and their Role in Cochlear and Vestibular Disorders*” delivers up-to-date knowledge on this Research Topic (Figure 1).

**Figure 1:**
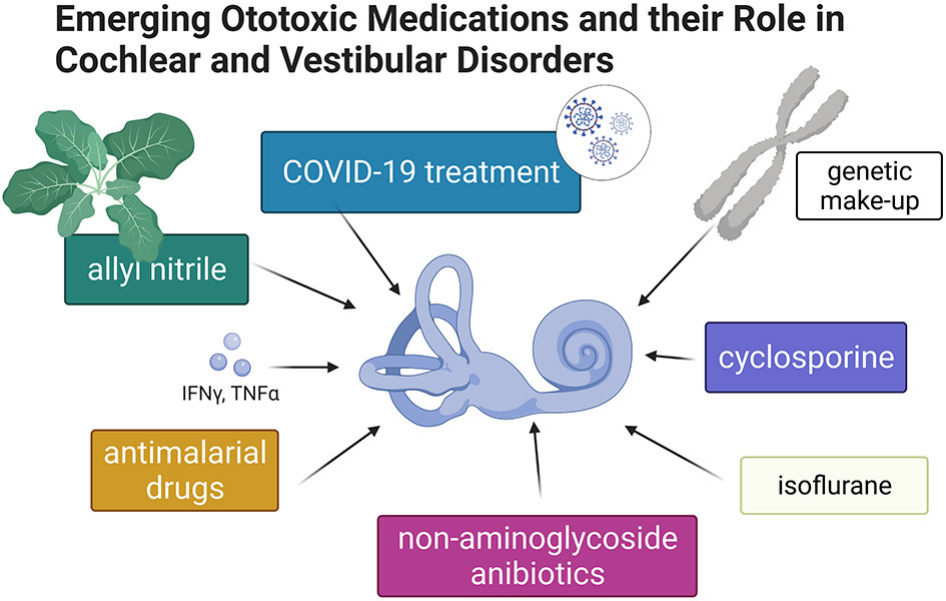
Graphic summary of topics discussed in this special issue of Frontiers in Neurology–Neuro-Otology (Created with BioRender.com).

In their report “The Ototoxicity of Antimalarial Drugs—A State of the Art Review,” Jozefowicz-Korczynska et al. carefully review the ototoxic properties of antimalarials. By analyzing papers published in the past 35 years, the authors conclude that the evidence for vestibulocochlear toxicity of antimalarials is still unclear. Nevertheless, the recent renaissance of dispensing antimalarials during the COVID-19 pandemic calls for awareness about the potential detrimental action of this class of drugs, supported by experimentally demonstrated death of mechanoreceptive hair cells in the lateral line of zebrafish induced by chloroquine phosphate or hydroxychloroquine (3).

Rybak et al. call our attention to the “Ototoxicity of Non-aminoglycoside Antibiotics.” This is a timely and fundamental subject because the widespread global use of antibiotics in humans and animals has created multidrug resistance among several microbial species (4), and the use of antibiotics continues to increase (5). The authors thoroughly review the field and present the evidence for ototoxicity of capreomycin, erythromycin, azithromycin, clarithromycin, vancomycin, and chloramphenicol. In addition, the authors define who is at the particular risk for ototoxicity induced by the above-listed medications.

The evidence supporting the existence of resident immune cells in the inner ear is accumulating (6). Therefore, it is not surprising that the ototoxic properties of cyclosporine—an immunosuppressor acting on lymphocytes, macrophages, and dendritic cells—were chosen by Sofia Waissbluth for a topic of an opinion paper, “Is Cyclosporine Ototoxic?” The author reviews reports concerning cochlear ototoxicity associated with cyclosporine administration in renal and liver transplant recipients and patients with nephrotic syndrome. Although the evidence is scarce, it still calls for clinical attention and more basic research in that field.

Coffin et al. focused on “Detecting Novel Ototoxins and Potentiation of Ototoxicity by Disease Settings.” Using COVID-19 pharmacotherapeutics as an example, the authors point to the ototoxic consequences of their intake. Interestingly, the authors also discuss the factors such as inflammation, kidney damage, and oxygen depletion as possible potentiators of drug ototoxicity. The authors further highlight the importance of human mitochondrial and genomic makeup that affect ototoxic outcomes of pharmacological treatments. Lastly, the authors present models for preclinical studies of ototoxicity.

In the paper “Protective Effects of Deferoxamine on Vestibulotoxicity in Gentamicin-Induced Bilateral Vestibulopathy Rat Model,” Kim et al. studied a method for protecting from aminoglycoside-induced ototoxicity in an animal model. The authors found that an iron-chelating agent given systemically, via an intramuscular injection, protects from the adverse effects of a locally administered aminoglycoside. Functional tests combined with the histopathological examination of the vestibular organs provided additional evidence for treatment efficacy. The primary mode of action is suggested to be iron chelation, preventing the gentamicin-iron complex formation and subsequent oxidative stress.

In the last paper published in this Research Topic, “Effect of Oral Allyl Nitrile Administration on Cochlear Functioning in Mice Following Comparison of Different Anesthetics for Hearing Assessment,” Verdoot et al. addressed two issues: one focused on allyl nitrile and the other focused on isoflurane and ketamine/xylazine. Allyl nitrile is not a drug but a substance, naturally occurring in cruciferous vegetables (e.g., cauliflower, cabbage, bok choy, broccoli), representing an important source for human consumption. One should consider possible toxicity when using a monotonous diet that includes these vegetables (7). In allyl nitrile-exposed mice, thresholds of auditory brainstem responses (ABR) and distortion product otoacoustic emissions (DPOAE) were permanently elevated, consistent with hearing loss. The vestibular function was not affected. When comparing two types of anesthetics—isoflurane vs. ketamine and xylazine combination–the authors found that isoflurane induced a significant threshold shift in DPOAE and ABR. This study delivers a critical warning to all researchers opting to use inhalation anesthetics for auditory experiments in mice.

When launching this Research Topic, we hoped to extend the knowledge about the ototoxicity of well-establish drugs and present the evidence for potential ototoxicity of relatively new drugs. The toxic properties of some of these drugs, including phosphodiesterase-5 inhibitors, antivirals, and synthetic opiates, have recently been described (8). However, little is known about the newly emerging and very dynamic class of medications–biologics. A recent review of pharmacological databases (9) has identified ototoxic properties of 194 medications—among them, five biologics: dasatinib (a protein tyrosine kinase inhibitor), erlotinib (an epidermal growth factor receptor inhibitor), recombinant interferon, muromonab (a monoclonal antibody targeted at the CD3 receptor), and pembrolizumab (a humanized antibody that blocks PD-1 located on lymphocytes). Dasatinib, interferon, muromonab, and pembrolizumab were reported to affect the cochlea and peripheral vestibular organs, whereas erlotinib was reported to cause cochlear toxicity. Clinical observations need to be complemented with a better understanding of the underlying mechanisms through basic and translational investigations on the ototoxic potential of biologics. Such research needs a significant shift in methods, including utilization of human cellular models of the inner ear *in vitro* (10) and humanized animal models *in vivo* (11), as biologics are highly human-specific.

Tuberculosis (TB) is an example of how causative microbes evolve, and how the therapy changes too. Notably, the WHO estimates that in 2021, about 25% of the world's population is infected with *Mycobacterium tuberculosis*—the bacterium causing TB. The incidence per 100 000 people varies from <10 in Europe to over 500 in the Philippines and 700 in sub-Saharan Africa. A multidrug-resistant (MDR) species of *M. tuberculosis* emerging in the last decades accounts for 3.4% of the new cases per year. Until recently, the treatment against that mutant involved intravenous aminoglycosides, resulting in hearing loss in every other patient (12). The WHO's recommendations on treating MDR TB have recently changed (13), and drugs other than aminoglycosides are now recommended instead, e.g., rifampicin, ethambutol, pyrazinamide, and levofloxacin. The duration of these combination therapies varies between 4 and 18 months. This dynamic change highlights the need for constant monitoring of possible adverse effects of the new and repurposed medications on hearing and balance functions. Our Research Topic draws attention to this need.

## Author Contributions

AS drew the figure. Both authors contributed to the article and approved the submitted version.

## Conflict of Interest

The authors declare that the research was conducted in the absence of any commercial or financial relationships that could be construed as a potential conflict of interest.

## Publisher's Note

All claims expressed in this article are solely those of the authors and do not necessarily represent those of their affiliated organizations, or those of the publisher, the editors and the reviewers. Any product that may be evaluated in this article, or claim that may be made by its manufacturer, is not guaranteed or endorsed by the publisher.
